# Investigation of Fluidic Universal Gripper for Delicate Object Manipulation

**DOI:** 10.3390/biomimetics8020209

**Published:** 2023-05-18

**Authors:** Changchun Wu, Hao Liu, Senyuan Lin, Yunquan Li, Yonghua Chen

**Affiliations:** 1 Department of Mechanical Engineering, The University of Hong Kong, Hong Kong, China; u3575077@connect.hku.hk (C.W.); u3575051@connect.hku.hk (H.L.); lsunkey@connect.hku.hk (S.L.); 2 Shien-Ming Wu School of Intelligent Engineering, South China University of Technology, Guangzhou 510006, China; yunquanli@scut.edu.cn

**Keywords:** soft robotics, universal grippers, variable stiffness, jamming transition, shear thickening fluid

## Abstract

The compliance of conventional granular jamming universal grippers is limited due to the increasing friction among particles when enveloping an object. This property limits the applications of such grippers. In this paper, we propose a fluidic-based approach for universal gripper which has a much higher compliance compared to conventional granular jamming universal grippers. The fluid is made of micro-particles suspended in liquid. Jamming transition of the dense granular suspension fluid from a fluid (hydrodynamic interactions) to solid-like state (frictional contacts) in the gripper is achieved by external pressure from the inflation of an airbag. The basic jamming mechanism and theoretical analysis of the proposed fluid is investigated, and a prototype universal gripper based on the fluid is developed. The proposed universal gripper exhibits advantageous compliance and grasping robustness in sample grasping of delicate objects, such as plants and sponge objects, where the traditional granular jamming universal gripper fails.

## 1. Introduction

To grasp objects of various sizes and shapes, robotic grippers based on different design principles have been developed by researchers. Compared with rigid humanoid grippers, soft universal grippers possess unique merits because of their brilliant compliance with the environment, which leads to fewer requests on structure design, control algorithms, and sensory feedback. In 2010, based on granular jamming (also called particle jamming) [1], a fluid-to-solid phase transition phenomenon, a soft universal gripper was proposed by Brown et al. [2]. With a simple structure of elastic membrane filled with granular materials, such a universal gripper is adaptive to different object shapes passively at the low stiffness state and freezes the shape at high stiffness when vacuumized. Objects with a wide variety of shapes, sizes, and weights can be grasped by this universal gripper. Since then, jamming grippers with various structures, such as snake-like structures [3] and multi-finger structures [4,5], have been developed by researchers. For such universal grippers, interparticle friction, geometrical interlock, and jamming force are the major factors that affect grasping performance. Grasping performance are mainly evaluated by compliance (applied force in enveloping an object) and grasping robustness (holding force or pull-off force) in general [2,6,7,8]. The effect of granular material properties, such as size, shape, and surface roughness, on grasping performance of universal grippers was also investigated [3]. It was suggested that ground coffee grains exhibit advantages both in compliance and jamming stiffness among many granular materials [2,3]. A partial-filled jamming universal gripper was found to have improved compliance in manipulating various living creatures under the deep sea [9]. However, the grasping robustness of this partial-filled universal gripper is adversely affected by its limited particle packing density. In contrast, Amend et al. proposed a universal gripper with refined grasping robustness and reduced compliance by applying positive pressure [7]. By integrating partial-filled and positive pressure schemes, an inflatable universal gripper demonstrating advantages in both compliance and grasping robustness was proposed by Wang et al. [8]. This universal gripper is fully filled initially and is inflated to partial-filled state to provide the gripper with a better compliance when enveloping an object. The gripper is then transformed back to a fully filled state by vacuum pressure to achieve good grasp robustness and rigidity. Though the partial-filled universal gripper and inflatable universal gripper exhibit relatively good compliance, their compliance is still suitable for grasping of only rigid or relatively fragile objects, such as raw eggs because the intrinsic jamming mechanism of these universal grippers is based on physical particle contact. Though conventional granular jamming is considered a phase transition from fluid-like state to solid-like state in general, the interactions among particles in both states are in fact hard frictional contacts, while in our proposed fluidic jamming method, hydrodynamic force dominates the interparticle interactions. The hydrodynamic force arises from the fluid particle velocity and acceleration, which is generally not at a high level when the gripper is grasping an object. Consequently, conventional granular jamming systems are hardly comparable to fluidic-based jamming in terms of compliance. In this paper, by utilizing a dense granular suspension fluid, a type of shear thickening fluid, we can achieve phase transition from a fluidic state (hydrodynamic interactions) to a solid-like state (frictional interactions) by applying an external pressure. This phenomenon has been tapped to develop a prototype universal gripper that demonstrates greater compliance than conventional jamming universal grippers. The superior compliance of the proposed universal gripper is shown in a number of grasping demonstrations of delicate objects such as plants, thin metal wire, sponge, and plastic drink package.

The basic principle behind the proposed research is based on shear thickening fluids, whose viscosity increases with the shear rate or shear stress and can recover spontaneously after the removal of external impact. Because of this unique property, shear thickening fluids have a wide variety of applications, such as shock absorption [10,11], human body protection [12], and surface polishing [13,14]. Shear thickening fluids can be mainly classified as granular suspensions, crosslinked polymer, micelle, and insoluble polymer systems [15]. Because a dense granular suspension (corn starch suspension) is applied in this study, we will only focus on granular suspensions in this research. Dense granular suspensions are suspensions of non-Brownian particles with high volume fraction immersed in a Newtonian solvent. When the particle size in a suspension exceeds 10 μm, the Brownian force in the suspension is neglectable; thus, contact forces (interparticle or external forces which may incur particle contact) only compete with hydrodynamic interactions, inducing easy formation of particle contacts. Hence, dense granular suspensions typically exhibit diverse and intriguing non-Newtonian behaviors, including shear thickening and jamming [16,17]. In shear thickening, the viscosity, or the resistance to the flow of a suspension, increases with shear rate. At low shear rates, the viscosity increases continuously with shear rate (continuous shear thickening), and the suspension exhibits considerable fluidity. On the premise that the volume fraction ϕ of the granular suspension is high enough, when the shear rate exceeds the critical point, a discontinuous transition occurs, and the viscosity of the suspension increases by orders of magnitude. As the precursor of fully jamming state, the suspension under discontinuous shear thickening (DST) demonstrates a transient and reversible transition from fluid to solid-like state. For jamming to occur, when the volume fraction of a granular suspension ϕ is higher than its particle-pressure-dependent jamming fraction $\phi_{j}$, the suspension cannot flow anymore, and the jamming transition can be observed [18,19,20]. If we consider that the particles immersed in a dense granular suspension are hard spheres, the jamming fraction $\phi_{j}$ of the suspension is relevant to the shape, polydispersity, and sliding friction coefficient of the particles [21]. For a granular suspension, the jamming volume fraction $\phi_{j}$ always ranges from $\phi_{rcp}=0.64$ for frictionless particles (random close packing fraction) to $\phi_{rlp}=0.55$ for particles with infinite frictional coefficient (random loose packing fraction) [22]. Because of the obvious fluid to solid transformation phenomenon, dense granular suspensions have become the most widely used shear thickening fluid and have been applied to personal protection devices [23,24]. However, shear thickening fluids have not been extensively explored in the robotic field owing to their poor controllability. In this study, we explore the controllable and sustainable jamming of a dense granular suspension fluid and apply it to a universal robotic gripper design for demonstration of its high compliance.

**Figure 1:**
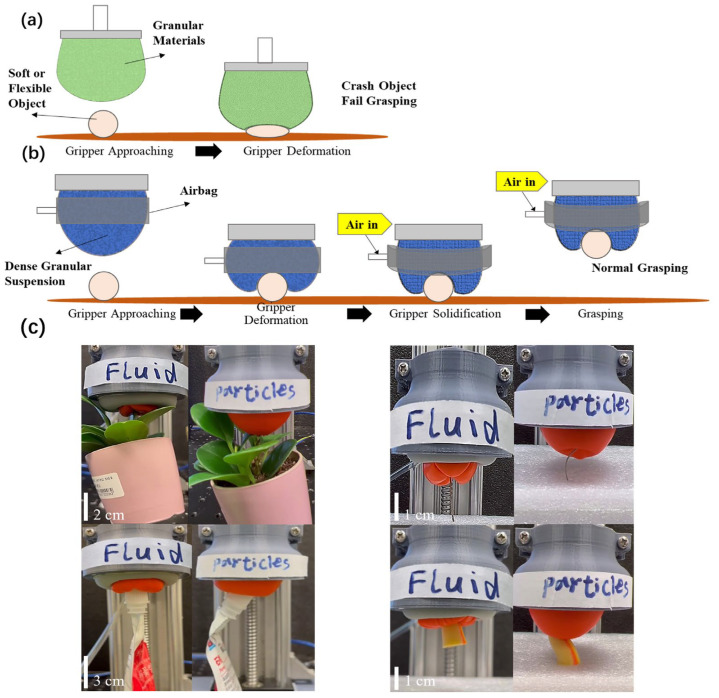
Universal gripper grasping comparison: (**a**) Conventional jamming universal gripper fails for soft object grasping. (**b**) Soft object grasping by the proposed universal gripper based on dense granular suspension fluid. (**c**) Sample soft/delicate object grasping using the proposed gripper for a pot of plant, a plastic sheet package of juice, a slim metal wire, and a foam earplug.

The complete grasping process of a conventional universal gripper involves approaching the object, deforming around the object, gripper solidification, and object manipulation [2]. Though conventional universal grippers demonstrate good performance in rigid object grasping, they may fail in grasping soft or delicate objects because the objects may not be able to stand the contact force, as shown in Figure 1a. In this paper, the universal gripper uses a dense suspension fluid; thus, the gripper has a better fluidity compared to direct dry particle contact. It can therefore envelop soft or delicate objects as long as they are more rigid than the fluid. Characterized with compliance reaching the fluid domain, the proposed universal gripper is capable of accomplishing grasping of general soft objects, as shown in Figure 1b, where jamming is caused by external pressure forming an airbag. Figure 1c is a comparison of sample object grasping where a conventional universal gripper (filled with 50 mL glass beads with 0.8–1 mm diameter) fails and the proposed universal gripper (50 mL corn starch suspension) excels. Detailed design of the universal grippers will be explained in subsequent sections. In the figure, four types of soft or delicate objects are used as examples: a pot of plants, a plastic juice package, a foam earplug, and a slim metal wire. It can be seen that the proposed universal gripper has no problem in enveloping the objects, while the conventional universal gripper will crush the objects. A video file will be supplemented to show the actual grasping process (Video S1). Furthermore, selected characteristics of the proposed universal gripper are experimentally evaluated, such as the test of pull-off force and applied force.

## 2. Result

### 2.1. Working Mechanism

#### 2.1.1. Jamming of Dense Granular Suspensions and Frictional Contact Network

**Figure 2:**
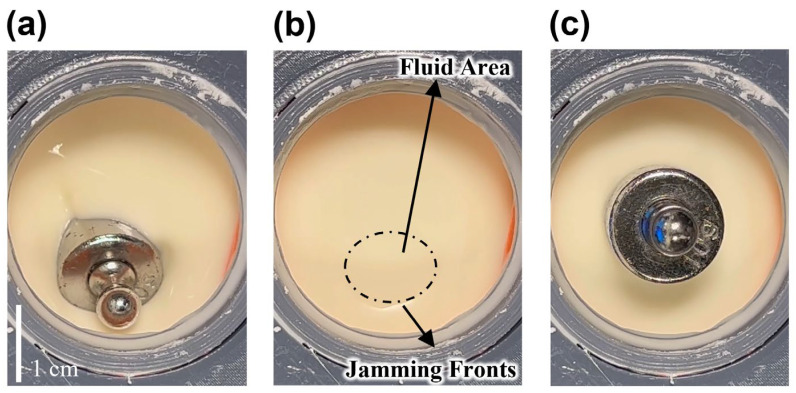
Fluid stiffening: (**a**) Fluid state of dense corn starch suspension fluid before jamming (10 g weight sinks into the suspension). (**b**) Jamming fronts propagate from side wall when external pressure is applied. (**c**) Fully jammed state of the dense corn starch suspension fluid (the weight stays on the surface of thickened fluid).

The phenomenon of a person being able to run on a dense corn starch suspension is long-documented [25]. Under impact, the formation of dynamic jamming fronts makes the dense corn starch suspension become transiently solidified and resistant enough to hold a person’s weight [18]. Furthermore, when the shear stress exerted is large enough, a dense granular suspension can get fully jammed by pure steady-state shear without any compression [19]. Either jamming induced by shear or compression, the increase of particle pressure, or more generally particle normal stresses, is the intrinsic cause of complete jamming for dense granular suspensions [16,17,19,20]. Wyart and Cates [20] proposed that there is a finite repulsive force between particles, whose range is far smaller than the particle radius. The existence of this repulsive force prevents the breakdown of the interparticle fluid film and the formation of hard contact between particles, and further influences the initiation of jamming or the value of jamming volume fraction. By microscopically measuring the interparticle force directly, Comtet et al. [26] presented experimental evidence proving the existence of such repulsive force between particles in granular suspensions. For a confined, dense, granular suspension with a volume fraction ϕ, when the particle pressure is low, the repulsive force between particles cannot be overcome, and thus the interparticle contact is lubricated (frictionless) by the fluid film. At a particle pressure sufficiently high to conquer the repulsive force, fluid films in the suspension begin to be compressed infinitely; hence, direct interparticle contacts appear and a contact network forms preliminarily. In the situation of rough particles, this direct interparticle contact is frictional, thus initially causing continuous or discontinuous augmentation in viscosity and the decrease of jamming fraction $\phi_{j}$ for the suspension. The jamming fraction $\phi_{j}$ of a dense granular suspension monotonically decreases with the fraction of interparticle frictional contacts in it. If the particle pressure continues to increase, the fraction of fluid films that have ruptured to form frictional interactions further increases as well until the constant volume fraction ϕ of the dense granular suspension is higher than its present jamming fraction $\phi_{j}$. In this condition, propagation of the frictional contact network reaches the whole confined volume of the suspension (may not involve all the particles). The frictional contact network in the suspension constrains the motion of particles immersed and can resist external force. As a consequence, the dense granular suspension is fully jammed and is characterized with a certain stiffness. This jamming transition is illustrated in Figure 2 using corn starch suspension fluid. Furthermore, if we consider $f_{c}$ as the fraction of frictional contacts in a dense granular suspension, complete jamming of the suspension can only be observed under the precondition that the volume fraction ϕ is higher than the boundary value $\phi_{m}$($\phi_{j}$ ($f_{c}$=1)). The boundary value $\phi_{m}$ is highly dependent on the frictional coefficient of the particles.

#### 2.1.2. Universal Gripper Based on the Jamming of a Dense Granular Suspension

As mentioned above, the dense granular suspension of volume fraction $\phi >\phi_{m}$ becomes fully jammed when ϕ is larger than its present particle-pressure-dependent jamming fraction $\phi_{j}$. According to the Wyart–Cates model [15], which neglects gravity and inertia, the jamming fraction $\phi_{j}$ can be described as a weighting function of the frictional contact fraction $f_{c}$, utilizing $\phi_{rcp}$ and $\phi_{m}$ as the weight factors ($\phi_{rcp}>\phi_{m}$):

(1) $$\phi_{j}\left(f_{c}\right)=f_{c}\phi_{m}+\left(1-f_{c}\right)\phi_{rcp}$$

The fraction of frictional contacts $f_{c}$ is expressed as a function of particle pressure:

(2) $$f_{c}\left(P\right)=1-e^{(-P/P_{r})}$$

**Figure 3:**
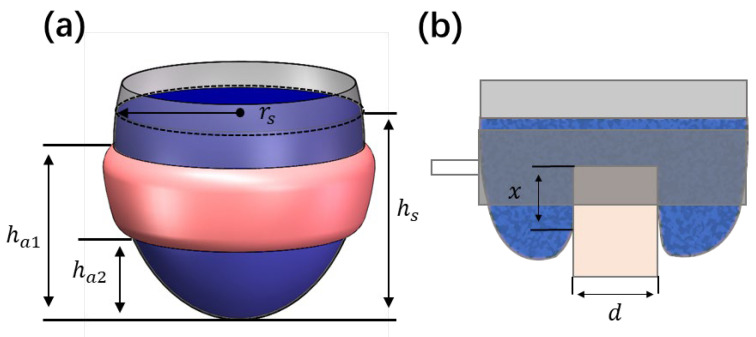
Gripper schematic: (**a**) Gripper dimensions. (**b**) Contact illustration of a sample cylindrical object.

**Figure 4:**
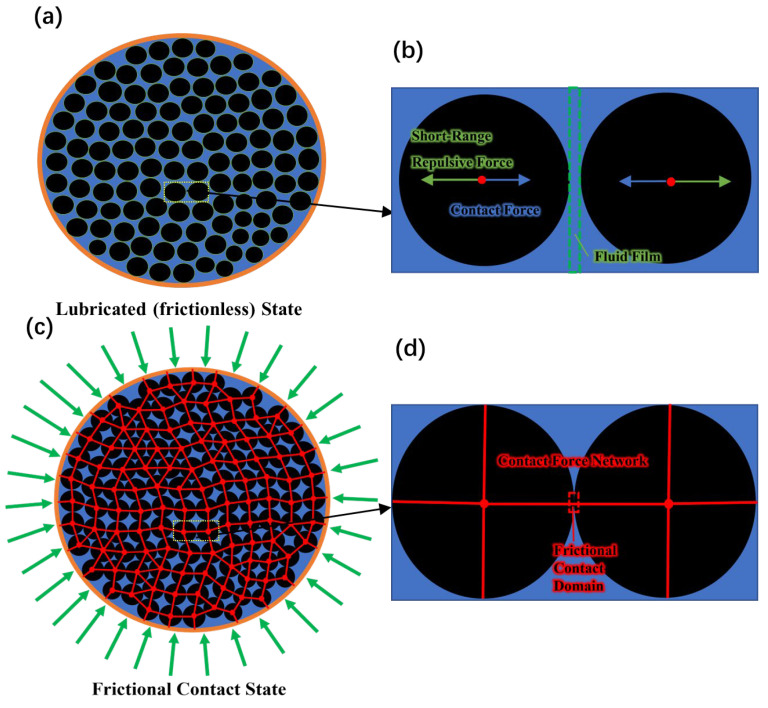
Jamming transition illustration: (**a**) Lubricated state of the dense granular suspension. (**b**) Short-range repulsive force prevents interparticle frictional contact. (**c**) Frictional contact network reaches the whole dense granular suspension by airbag pressure. (**d**) Fluid film is ruptured to form interparticle frictional contact.

In Equation (2), P is particle pressure in the suspension, $P_{r}$ represents the interparticle repulsive force that protects the fluid film. The strength of $P_{r}$ is relevant to the critical particle pressure that causes shear thickening to occur. Based on Equations (1) and (2), the jamming fraction $\phi_{j}$ monotonically decreases with the particle pressure P; hence, increasing the particle pressure is the key to induce the jamming of a dense granular suspension of volume fraction $\phi >\phi_{m}$. As shown in Figure 3a, for the universal gripper proposed in this study, an annular airbag is used to increase the particle pressure of the dense granular suspension fluid inside the membrane. The dense granular suspension contained in the membrane is squeezed by the inflated airbag, resulting in a pressure increase of the suspension fluid. In a grasping operation, as the gripper deforms around the object in a fluid state as shown in Figure 4a,b, particles are immersed in the solvent and lubricated by the fluid film due to the existence of interparticle repulsive force. Thus, particles have no direct contact. The viscosity of the dense granular suspension fluid can be utilized as the main quantitative indicator to evaluate the performance of the proposed universal gripper in the fluid state. Lower viscosity indicates better compliance with objects and lower applied force in enveloping an object. In accordance with the Wyart–Cates model [20,22] and Krieger–Doughtery relation [27,28], for a dense granular suspension fluid of volume fraction $\phi <\phi_{j}$, the viscosity can be expressed as a function:

(3) $$\eta =\frac{\eta_{0}}{{\left(1-\phi /\phi_{j}\right)}^{2}}$$

where η is the viscosity of the dense granular suspension, $\eta_{0}$ is the suspending solvent viscosity. As Equations (1) and (2) shown, $\phi_{j}$ is inversely proportional to particle pressure P, and P at a fixed volume fraction $\phi <\phi_{j}$ satisfying the relation:

(4) $$P\propto \eta \dot{\gamma }$$

where $\dot{\gamma }$ represents the shear rate. As a consequence, the viscosity decreases with the shear rate. For our proposed universal gripper, the enveloping stage in grasping is in the fluid state. In general, the compliance decreases as the enveloping around the object progresses. Moreover, the gravity of the suspension fluid and elasticity of the membrane are two impact factors of the gripper’s compliance that need more in-depth studies in future. During grasping, the gripper is solidified after adaptive enveloping of an object is complete and the object is securely held. The solidification of the gripper is achieved by increasing air pressure inside the airbag to a level where accumulative fluid films rupture and the frictional contact chain propagates until covering the entire volume of the dense granular suspension. The jamming transition and solidification of the gripper is illustrated as shown in Figure 4c,d. For the proposed universal gripper, it is important to determine the specific air pressure that induces the jamming transition or gripper solidification. Thus, the relationship between the air pressure of the airbag and the particle pressure in the suspension fluid needs to be discussed. Since the airbag is not fully inflated due to constraint from the gripper shell and suspension volume, the internal air pressure of the airbag can be considered as the same as the pressure exerted on the suspension volume directly. Air pressure is distributed so that the particle pressure in the suspension fluid that is covered by the airbag approximates the airbag’s air pressure [29]. Let the airbag contacted surface area $S_{a}=S_{s}$ (the surface area of granular suspension), the particle pressure (in the entire granular suspension inside the gripper) P is dependent on the airbag’s air pressure $P_{a}$ [29]. The airbag covering surface area can be obtained by $S_{a}=\pi r_{s}({h_{a1}}^{2}-{h_{a2}}^{2})/h_{s}$ and surface area of the granular suspension is $S_{s}=\pi r_{s}h_{s}$. Hence, the relation between P and $P_{a}$ can be expressed as:

(5) $$P=\alpha {\left(\frac{S_{a}}{S_{s}}\right)}^{\beta }P_{a}=\alpha {\left(\frac{\left({h_{a1}}^{2}-{h_{a2}}^{2}\right)}{{h_{s}}^{2}}\right)}^{\beta }P_{a}$$

where α (α < 1) and β are found from experimental data. By integrating Equations (1), (2) and (5), we can predict the onset of fully jamming of the dense granular suspension inside the gripper induced by airbag pressure. Some parameters in these equations, such as $\phi_{m}$, $P_{r}$, α, and β, will be further discussed and refined by experimental data for more accurate modeling.

Holding force $F_{h}$ can be employed to evaluate the performance of the gripper in the solid state. For grasping objects of irregular shape, the holding force $F_{h}$ can be amplified due to the interlocking phenomenon. To simplify the modeling and enhance the reliability of judging whether the gripper can successfully grasp an object based on the calculated holding force $F_{h}$, the geometric interlocking phenomenon is neglected here. Therefore, only the friction mechanism $F_{f}$ contributes to the gripper’s holding force $F_{h}$, and the relation $F_{h}=F_{f}$ can be obtained. If we consider the object has no sliding and deformation during grasping, the effective contact area between the membrane and the object remains constant and can be used as the shear area $A_{s}$. For the cylindrical sample object shown in Figure 3b, the shear area $A_{s}=\pi dx$. Since the dimension of particles immersed in the suspension is far smaller than the dimension of the object grasped, the contact area between the gripper and the object has a distributed pressure of P. Then, the normal force $F_{n}$, has the expression $F_{n}=PA_{s}$. In accordance, the friction force $F_{f}=\mu_{c}F_{n}$, where $\mu_{c}$ is the friction coefficient of the contact area, and combined with Equation (5), we can illustrate the relation between holding force and input airbag pressure as:

(6) $$F_{h}=\mu_{c}\alpha {\left(\frac{\left({h_{a1}}^{2}-{h_{a2}}^{2}\right)}{{h_{s}}^{2}}\right)}^{\beta }P_{a}A_{s}$$

From Equation (6), we can see major factors that have effects on holding force or performance of the proposed universal gripper in solid state, such as dimension of the airbag, roughness of membrane (coefficient of friction), the input airbag pressure, and contact area with the object. Additionally, for object grasping with interlock mechanism, the gripper must deform before an object is pulled off; thus, the force inducing gripper deformation is considered as a part of the holding force. The force required for gripper deformation is related to gripper stiffness, which is generally associated with particle friction coefficient $\mu_{p}$ (as in the conventional jamming case). Thus, for the object grasping with geometric interlock, the particle frictional coefficient $\mu_{p}$ should be taken into consideration as a factor relevant to the gripper holding force.

### 2.2. Experimental Characterization

**Figure 5:**
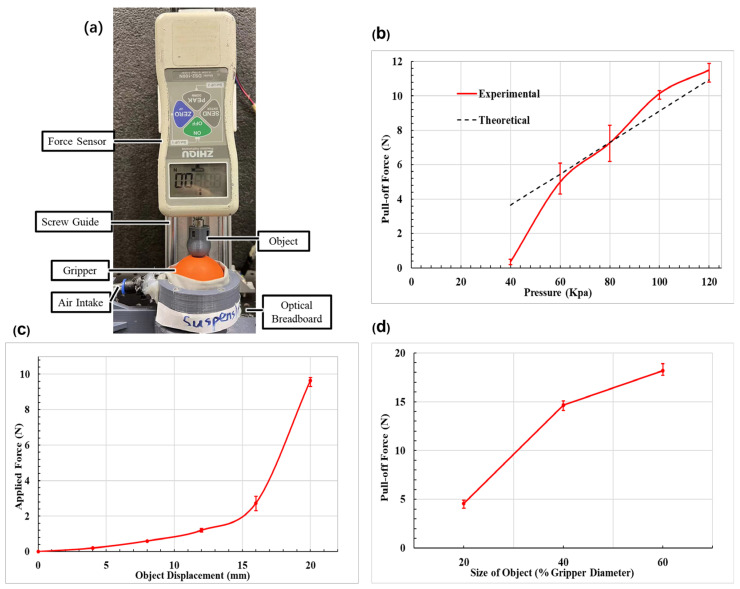
Grasping performance tests: (**a**) Experimental Set up. (**b**) The pull-off force versus input air pressure. (**c**) The applied force versus object displacement. (**d**) The pull-off force versus size of object.

To characterize the performance of the proposed universal gripper, a number of experiments have been conducted using the experimental platform as shown in Figure 5a. The proposed universal gripper is fixed on a table. Sample objects are installed on the probe head of a force meter which can measure both compression and tension. The sampling frequency and resolution of the force sensor are 1000 Hz and 0.1 N respectively. The force meter is mounted on a vertical linear screw guide whose displacement is recorded by a data terminal. Three sets of tests, pull-off force versus input pressure of the airbag, pull-off force versus the size of objects, and applied contact force versus displacement, are accomplished using the experimental platform. The pull-off force indicates the force required to separate the object from jammed gripper and the applied force is the force required to deform the gripper to certain extent so that the gripper may envelop the object. Each test is repeated three times and the average is used take for the plot in the figures. 

From Figure 5b, it is observed that the pull-off force is proportional to the airbag input pressure and reaches 11.5 N at 120 kPa input pressure. The theoretical result can depict the relation between pull-off force and input pressure (α=0.064,β=0.6471). In these tests, the object is spherical, and its size is ϕ 21.5 mm (50% of gripper diameter), and the displacement of the object inserted inside the gripper is 20 mm. As shown in Figure 5c, the applied force of the proposed universal gripper is obviously very small in the initial stage of enveloping an object (before 15 mm insertion depth). The applied force increases dramatically after 15 mm insertion displacement. This might be caused by the increasing membrane tension that starts to trigger the fluid to stiffen. Before the membrane tightens, the proposed gripper demonstrates applied forces of less than 2 N. To be mentioned, though the inflatable universal gripper [8] adopts a partial-filled scheme to reduce the applied force, its applied forces still range from 6 N to 35 N. The pull-off force versus different sizes of objects is also tested. We employ objects of cylindrical shape with various diameter, including ϕ8.6 mm (20% of gripper diameter), ϕ17.2 mm (40%), and ϕ25.8 mm (60%) for the experiments. As shown in Figure 5d, though it is observed that the pull-off force increases with the size of object, the increase ratio decreases as the object size increases. When the object size is big enough that the gripper cannot properly form a good contact, the pull-off force might drop significantly. 

**Figure 6:**
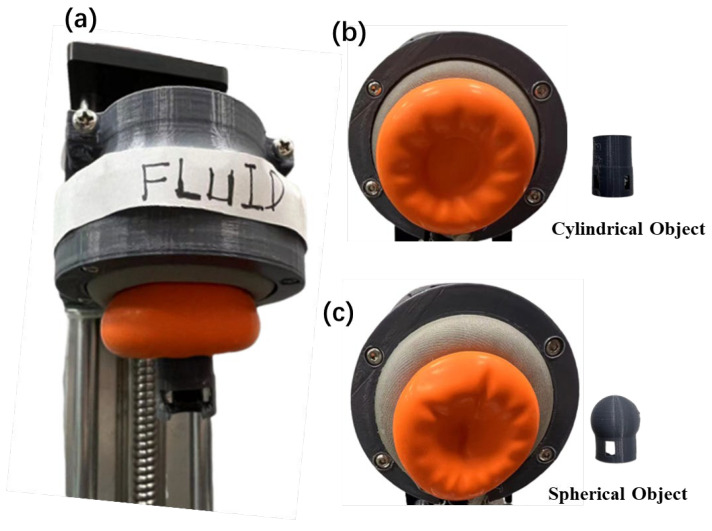
Gripper solidification due to jamming: (**a**) Successful grasping of the target object. (**b**) Maintaining the deformed profile after pulling off the cylindrical object. (**c**) Maintaining a spherical profile after pulling off the spherical object.

Finally, to demonstrate the capability of the proposed gripper in grasping soft and delicate objects, sample object grasping operations as shown in Figure 1c were conducted. A video file showing the actual grasping processes has been supplemented in this paper (Video S1). To demonstrate that the jamming transition is the primary mechanism behind the successful grasping of the proposed universal gripper, gripper deformed states are shown after grasping of different objects as shown in Figure 6. The gripper keeps the deformed state even after the objects are pulled off, as long as the air pressure is still on. This phenomenon is impossible for Newtonian fluids.

## 3. Material and Method

**Figure 7:**
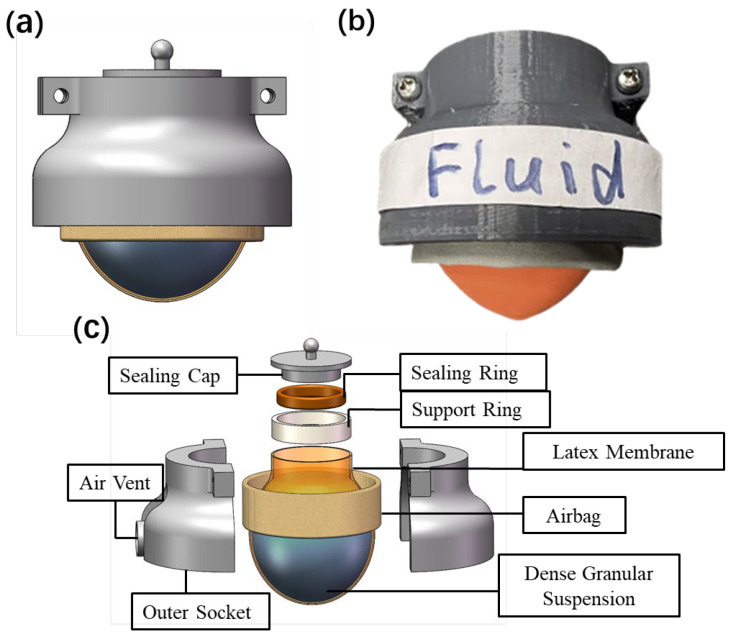
A prototype universal gripper: (**a**) Assembled design. (**b**) Prototype. (**c**) Exploded view of the design.

Figure 7a,b shows the design and prototype respectively. An exploded view showing every part in the design is also shown in Figure 7c. The prototype universal gripper consists of a membrane containing a dense granular suspension fluid, an airbag, an outer socket, and the sealing apparatus (sealing cap, sealing ring, and support ring). The membrane used is a latex balloon which is readily available. The airbag is fabricated with TPU-coated polyester fabric. The 3D-printed outer socket is used for fixing the membrane and the airbag, locating the air vent, and connecting other movable devices. The dense granular suspension utilized is a corn starch suspension fluid, with a given amount of cornstarch (Corn Starch, SELECT, Thailand) and a given amount of water. The volume fraction, or the ratio between the volume of cornstarch particles and the total volume: $\phi ={(m}_{cs}/\rho_{cs})/(m_{cs}/\rho_{cs}+m_{water}/\rho_{water})$, where the density of corn starch and water is $\rho_{cs}=1.55g/{cm}^{3}$ and $\rho_{water}=1g/{cm}^{3}$ respectively [26,30]. For the universal gripper used for sample grasping demonstrations in this study, the diameter of the gripper filled with 50 mL corn starch suspension is 43 mm, the height the of airbag is 23 mm, and the corn starch suspension ratio is of 51.5% volume fraction. To pursue a better compliance, the 50 mL corn starch suspension does not fully fill up the latex membrane, occupying around 80% of the initial volume of the membrane. This gripper has demonstrated good compliance in grasping soft or delicate objects, as has been shown in previous figures and the Supplementary Video S1. Even though the volume fraction of 51.5% is used for the prototype gripper design with good performance, the critical volume fraction is not yet theoretically modelled for the design. The critical volume fraction $\phi_{m}$ will be investigated in our future studies. 

## 4. Conclusions and Discussion

A universal gripper based on the jamming of a dense granular suspension fluid for soft and delicate object grasping is proposed and investigated in this study. We demonstrate that the positive-pressure-induced jamming transition of a dense granular suspension enables the proposed universal gripper to exhibit an obvious advantage in compliance. The mechanism of the jamming process of a dense granular suspension is also illustrated, and the required input pressure for the onset of fully jamming is estimated. For the proposed universal gripper, the combination of friction and geometrical interlocking mechanism contributes to the gripper holding force. The gripper holding force is dependent on several factors, such as air input pressure, gripper construction (the volume of suspension and the airbag covering surface area), size of object (contact area), and surface roughness (friction coefficient). Moreover, experimental studies, including pull-off force test and applied force test, are conducted to evaluate and investigate the gripper performance. From the results, the proposed universal gripper exhibits good performance that conforms to our expectations.

Compared with the traditional universal gripper, the universal gripper based on the jamming of a dense granular suspension may have a variety of applications that have requirements for high adaptability, such as grasping soft objects of different sizes and shapes. Though the proposed universal gripper shows promising performance and application prospects, some problems still need to be further studied for practical use. The adverse effect of the membrane elasticity on the gripper compliance should be minimized. Problems associated with the inherent properties of dense granular suspensions, such as dehydration in a non-hermetic environment and particle settlement for suspensions like the corn starch suspension still cause degrading performance over time. Vibration or adjunction of cesium chloride may solve or retard the settlement of corn starch suspension, and thus may partly solve these problems [19,22]. Furthermore, more efficient methods for inducing jamming of a dense granular suspension fluid also should be investigated. Shearing or vacuuming may have a similar effect to positive pressure. We expect that more interest in the jamming of dense granular suspensions for soft robotic research could be stimulated through this study.

## Data Availability

All data needed to support the conclusions of this manuscript are included in the main text or the Supplementary Materials.

## Supplementary Materials

The following supporting information can be downloaded at: https://www.mdpi.com/article/10.3390/biomimetics8020209/s1, Video S1: Grasping Demonstration of Fluidic Universal Gripper.
